# An output-based measurement of EU bioeconomy services: Marrying statistics with policy insight

**DOI:** 10.1016/j.strueco.2021.10.005

**Published:** 2022-03

**Authors:** Tévécia Ronzon, Susanne Iost, George Philippidis

**Affiliations:** a European Commission, Joint Research Centre (JRC), Seville, Spain.; b Agricultural Economics and Rural Policy Group, Wageningen University, the Netherlands; c Thünen Institute of International Forestry and Forest Economics, Hamburg, Germany; d Aragonese Agency for Research and Development (ARAID), Centre for Agro-Food Research and, Technology (CITA), Agrifood Institute of Aragón (IA2), Government of Aragón, Zaragoza, Spain

**Keywords:** Bioeconomy, Service, Value added, Employment, Productivity, Europe

## Abstract

In its revised bioeconomy strategy, the European Union (EU) has extended the scope of activities to include services. Employing an output-based approach, this study quantifies the contribution of bioeconomy services to gross domestic product and employment in the EU Member States over 2008–2017. Moreover, it also identifies the main sectoral sources of employment and growth within bioeconomy services. The choice of Eurostat statistics ensures data harmonisation across countries and continuity for future updates, although important data needs are identified to enhance the representation of bioeconomy services within European statistical frameworks. In 2015–2017, economic growth was stronger in bioeconomy services than in the total EU economy. Bioeconomy services accounted for between 5.0–8.6% and 10.2–16.9% of EU gross domestic product and the EU labour force, respectively, whilst three service sectors account for more than 60% of bioeconomy services employment and value added. Interestingly, in the decade up to 2017, labour productivity in bioeconomy services improved.

## Highlights

• Bioeconomy services are gaining importance within European bioeconomy strategies.
• We calculate the employment and value added size of European bioeconomy services.
• The methodology focuses on services within the system of national accounts.
• They showed stronger economic growth than the overall European economy in 2015–2017.
• 60% of bioeconomy services' size comes from wholesale, retail trade and food services.

## 1 Introduction

The first bioeconomy strategy launched by the European Union in 2012 defined the bioeconomy as ”the production of renewable biological resources and the conversion of these resources and waste streams into value added products” (European Commission, 2012 p. 3). The strategy supported a higher sourcing of production processes with renewable biological resources and encouraged cascading uses of biomass, bio-based products and bio-based waste streams along pre-existing and novel value chains. In addition, targeted support to research and innovation aimed at bolstering the development of new bio-based products to realise a transition towards a low-carbon economy with the associated benefit of offering new market opportunities to biomass suppliers (i.e., farmers, foresters and fishers). Since 2012, the bioeconomy in Europe has gradually enhanced its credentials as a 'green growth strategy' by broadening its sphere to encompass related services activities and by integrating the notion of environmental preservation. Indeed, this more comprehensive conceptualisation is reflected in the EU's revised definition, where the bioeconomy ”includes and interlinks: land and marine ecosystems and the services they provide; all primary production sectors that use and produce biological resources (…); and all economic and industrial sectors that use biological resources and processes to produce food, feed, bio-based products, energy and services” (European Commission, 2018 p. 4).

The bioeconomy - in its revised definition - has become instrumental in recent EU policies, for example, for the realisation of the Circular economy action plan (International Advisory Council on Global Bioeconomy, 2020), the blue (bio)economy, the forestry strategy and for the definition of the national strategic plans of the new Common Agricultural Policy (CAP). Moreover, due to its broad sectorial coverage, the bioeconomy is also pivotal for implementing the objectives of the Green Deal. Thus, the emergence and growth of the bioeconomy on the EU policy agenda triggered the need for an appropriate measurement of its size and dynamic as a basis for monitoring and impact assessments (Wesseler and von Braun, 2017).

The international system of national accounts (SNA) and its European equivalent, the ‘European System of Accounts’ (ESA), are the natural frameworks for economic measurement, monitoring and international comparison. They allow for a harmonised quantification of the emblematic growth indicator of gross domestic product (GDP), of which its statistical components serve as a basis for measuring total factor productivity (TFP). As a cornerstone of macroeconomic analyses, the SNA has adapted as economic knowledge and theory has evolved. In 1957, the Solow model of growth first proposed a decomposition of economic growth into the contribution of labour and capital inputs plus a residual described as TFP (Solow, 1957). Subsequent growth accounting approaches have sought to reduce this residual in order to better analyse the sources of economic growth and quantify the respective contribution of factor inputs and TFP (Jorgenson, 2018; Landefeld, 2020). These approaches integrate the KLEMS growth and productivity satellite accounts into the ESA, in which inputs are distinguished between capital (K), labour (L), energy (E), materials (M) and service inputs (S) at the industry-level (Eurostat, 2013 p. 502). The KLEMS accounts are closely linked with input-output tables, and with non-SNA complementary data sources (e.g. the Eurostat Labour Force surveys) (Koszerek et al., 2007). In sum, the joint development of economic models and the statistical framework has permitted detailed analysis on the sources of economic growth and the cross-comparison of growth trajectories (e.g. for the EU, Van Ark and Jäger, 2017; Hartwig and Krämer, 2019; Gordon and Sayed 2020; Inklaar et al., 2020; Jia et al., 2020).

The application of these theoretical growth models to conduct an analysis of the drivers of bioeconomy growth is, unfortunately, severely hampered by data gaps. More specifically, the standard international classifications of economic activities associated to the SNA and ESA frameworks are inadequate for the representation of bioeconomy activities, as many traditional and nascent bio-based industries cannot be singled out from the SNA industry categories. Consequently, the reconstruction of harmonised statistics on bioeconomy activities constitutes the very first and primordial step before any economic analysis. To meet this data need, the Joint Research center (JRC) of the European Commission has been steadily conducting quantification work on EU bioeconomy developments (M'barek et al., 2014) and most recently launched a bioeconomy monitoring system (Robert et al., 2020a). Methodologies for monitoring the more recent elements of the EU bioeconomy strategy are, however, not yet fully consolidated (i.e., bio-based services, ecosystem services, and the ecological boundaries of the bioeconomy).

From a policy perspective, eleven countries in the world plus the Nordic Council of Ministers and the EU mention the provision of services in their bioeconomy strategic documents (German Bioeconomy Council, 2018; International Advisory Council on Global Bioeconomy, 2020). But, bioeconomy services remain conspicuously absent from published economic analyses on the bioeconomy. This very same point is made in Capasso and Klitkou (2020, Section 4.2) who stress the limitation of neither considering services nor public administration in measurements in the European bioeconomy (i.e., Ronzon and M'Barek 2018), thus biasing downward the size of the bioeconomy with respect to the rest of the economy. Therefore, the aim of this paper is to construct reliable harmonised metrics of the bioeconomy services for the Member States of the EU and to provide a preliminary assessment of the performance of said sector. The paper is structured around three research questions: (i) how to define and quantify a bioeconomy service? (Sections 2 and 3), (ii) how does the aggregate of bioeconomy services sectors perform within the economy? (Section 4.1) and (iii) what are the sectoral sources of employment and wealth creation within bioeconomy services at the EU and Member State levels? (Sections 4.2 and 4.3).

Our analysis is based on the quantification of three key indicators: value added at the industry-level (or NACE level), employment in number of persons and labour productivity. The economic growth of bioeconomy services is measured with the growth rate of the aggregated value added for all bioeconomy services sectors; the number of persons employed gives an indication of labour inputs; and the labour productivity is calculated as the ratio of these two concepts. Indeed, in the absence of data on the productivity of capital inputs or on TFP, one cannot apply the aforementioned models of growth, whilst labour productivity remains the only indicator of productivity that one can reconstruct.

To ensure a consistent and replicable application over time and across the 27 EU Member States, this research proposes an SNA-compatible methodology in the sense that it follows the NACE classification (Eurostat, 2008) and is principally based on Eurostat statistical data. As a result, the outcomes from this research provide an evidence-based platform for tailoring policy coherent initiatives by Member State.

## 2 Defining the study's quantification approach

**Fig. 1:**
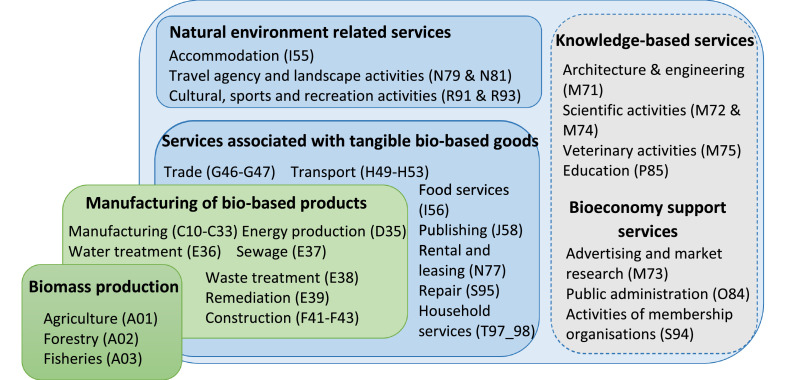
Categorisation of the bioeconomy activities within the NACE classification Note: The glossary in the supplementary material S1 provides full details on the activities represented here by their NACE code.

In the NACE classification, service industries are represented by the divisions G to T. This study, therefore, focuses on NACE G to T services that match the European Commission (2018) definition of activities based on the ‘use [of] biological resources and processes to produce (…) services’ or on the services provided by land and marine ecosystems (dark blue frames on Fig. 1). The latter include some marketed ecosystem services (e.g., nature accommodation or 'forest-based recreation, sports, and outdoor activities, and educational activities that are not free of charge to the users' (FOREST EUROPE, 2020)). The valuation of non-marketed or extra-NACE ecosystem services is beyond the scope of this study.

The NACE divisions G to T also embed upstream bioeconomy services supply chains such as research activities or the elaboration and implementation of bioeconomy strategies by public administration (grey frame on Fig. 1). In our interpretation, such activities do not directly match the European Commission (2018) definition of bioeconomy activities which rather targets ecosystem services and biomass using services. They are however, prominent in some EU Member States' bioeconomy scope, which justifies their inclusion within our methodology (Sections 3.4 and 3.5).

Note, that even enterprises producing or processing biomass (NACE A to F represented by the green frames on Fig. 1) may produce some services such as forest management, within-firm R&D and many others (see the overlap between the green and light blue frames on Fig. 1). These services are beyond the scope of the present study in order to avoid any double counting between the service activities (NACE G to T) and the non-service activities (NACE A to F).

There are different possible interpretable measurements of a bioeconomy service. The so-called “input-based approach” quantifies a bioeconomy service in proportion to the biomass-based inputs it uses. For example, in Robert et al. (2020b) bioeconomy publishing activities are measured in proportion to the sectors' usage of forestry, wood, paper and paper printing in total input uses from Eurostat Supply and Use tables (SUTs). Efken et al. (2016) quantify biomass input shares from cost structure statistics for services in Germany, except restaurants that are considered fully part of the bioeconomy. Kuosmanen et al. (2020, Section 4) propose the use of Input-Output table (IOT) data on agriculture, forestry and fishing inputs to all economic services as a proxy for biomass input shares.

Also employing IOT data, Cingiz et al. (2021) distinguish between a downstream and an upstream component in bioeconomy services. Similarly to the input-based approaches, the downstream component refers to the use of inputs from sectors considered fully belonging to the bioeconomy, namely agriculture, forestry, fishing, the printing industry and the manufacture of food, beverage, tobacco, wood products and paper. The upstream component refers to the provision of inputs to fully bioeconomy activities. In contrast with input-based approaches, the upstream component therefore includes into the bioeconomy size those service activities that use little or no biomass but contribute significantly to the input composition of fully bioeconomy sectors (e.g., banking, financial or technical advisory services). Note that a NACE sector can combine the downstream and upstream components. For example, wholesale and retail trade activities (G45–47) both use inputs from agriculture (agricultural commodities for sale) and source the agricultural sector.

Other approaches restrict the scope of bioeconomy services to the ones that match the EU or national definition of the bioeconomy. These approaches are sometimes called 'output-based' in the sense that the bioeconomy nature of a service is evaluated on the outputs' characteristics instead of on the inputs it uses. For example, Iost et al. (2019) fully include biotechnology research into their bioeconomy scope - no matter the proportion of biomass inputs used by this activity - because of the prominent place of biotechnologies in the German bioeconomy definition. As another example, the Finnish statistics define bioeconomy services as nature tourism and recreation activities as well as recreational hunting and fishing (Luke 2020). Their quantification is independent of the biomass input used by these activities, but it is derived from data on accommodation and catering in the case of nature tourism and recreation activities, and from the 'non-market output' of hunting, fishing, and aquaculture for recreational hunting and fishing.

Input approaches (or similar) offer a clear and 'systematic' measure of bioeconomy services that is applicable and harmonised across all sectors of the economy when applied to IOTs or SUTs. Rather than this systematic approach to measurement, output approaches tend to respect the specificities of each stakeholder's definition of the bioeconomy in line with adopted policy-priorities. The precision of the output method is very much dependant on data availability and on the level of data disaggregation available to determine the bioeconomy nature of services' outputs.

Following the discussion in the introduction, this study adopts a policy-driven measurement of bioeconomy services, thereby favouring an output-based approach aligned with the EU and Member States' definition of the bioeconomy. For the first time, it permits the application of a comprehensive policy-orientated approach of bioeconomy services across EU Member States.

## 3 Methodology and data

### 3.1 Overall quantification approach

This study quantifies value added (variable 'VA', expressed in million €), employment (variable 'E', expressed in number of people employed) and labour productivity (variable 'P', in thousand € per person employed) in the bioeconomy services that are reported under the NACE divisions G to T in Eurostat statistics. The main challenge lies in the determination of a bio-based output share b_n,c,y_ for adjusting official statistics to the measure of bioeconomy services only, where *n* denotes the NACE division level, *c* the country (i.e., the EU27 and the 27 Member States) and *y* the year (*y* = 2008, …, 2017) (see Sections 3.2 to 3.5).

Bio-based output shares b_n,c,y_ are retrieved from biomass contents published in the scientific literature or otherwise derived from Eurostat statistics. In the latter option, the precision in the quantification of b_n,c,y_ depends on the granularity of statistical data available. Bio-based output shares are preferably determined at the NACE sub-division level data *m* (3- or 4-digit code) and then aggregated to the 2-digit NACE level *n*, that is:

(1) $$•\;\text{Calculated}\;\text{on}\;\text{value}\;\text{added}\;\text{data}:{\;b}_{n,c,y}^{\text{VA}}=\frac{\sum_{m}\left(b_{m,c,y}\times VA_{m,c,y}\right)}{VA_{n,c,y}}$$

(2) $$•\;\text{Calculated}\;\text{on}\;\text{employment}\;\text{data}:{\;b}_{n,c,y}^{E}=\frac{\sum_{m}\left(b_{m,c,y}\times E_{m,c,y}\right)}{E_{n,c,y}}$$

Note that the employment distribution across sub-divisions differs from the value added distribution, leading to different b_n,c,y_ when calculated on employment data versus on value added data:

(3) $$\frac{\sum_{m}\left(b_{m,c,y}\times \;VA_{m,c,y}\right)}{VA_{n,c,y}}\neq \frac{\sum_{m}\left(b_{m,c,y}\times E_{m,c,y}\right)}{E_{n,c,y}}$$

It follows (where the prefix bb of each indicator refers to its ‘bio-based’ component):

(4) $$\text{bbV}A_{n,c,y}=b_{n,c,y}^{\text{VA}}\times \;VA_{n,c,y}$$

(5) $$\text{bb}E_{n,c,y}=b_{n,c,y}^{E}\times E_{n,c,y}$$

such that the productivity measure is given as:

(6) $$\text{bb}P_{n,c,y}\;=\text{bbV}A_{n,c,y}/\text{bb}E_{n,c,y}$$

Given the availability of NACE disaggregation, panel data for variables VA and E are taken from Eurostat structural business statistics (column 3 of Table 1, Eurostat (2020a, 2020b)). Moreover, Eurostat national accounts are employed to complement the NACE divisions not represented in the Eurostat structural business statistics (column 3 of Table 1, Eurostat (2020 g, 2020h)). The observation period runs from the most recent NACE classification revision (2008) to the latest available year (2017). Note that value added is reported in nominal prices in these data sources.

**Table 1:** Assumptions and data sources for the quantification of sectoral output bio-based shares, value added and number of persons employed in the bioeconomy (sector *n*, country *c*, year *y*).

NACE division - short name	Range of bio-based output shares b_n,c,y_ and/or b_m,c,y_ or literature source (‘n’ denotes 2-digit NACE codes, `m’ denotes 3- or 4-digit NACE codes)	Eurostat source for value added VA_n,c,y_ & employment E_n,c,y_
G45 – trade/repair vehic.	0%	
G46-H53 - Trade and transport	Product bio-based shares from nova-Institute*, otherwise 0–100%	sbs_na_dt_r2, sbs_na_1a_se_r2
I55 - Accommodation	Eurostat tour_occ_ninatd for I551-I553 (Section 3.3)	sbs_na_1a_se_r2
I56 - Food services	100%	sbs_na_1a_se_r2
J58 - Publishing	0% J582,	sbs_na_1a_se_r2
	0–100% J5811-J5814 and J5819	
J59-M70 – ICT, finance…	0%	–
M71 - Architecture and engineering	Same bio-based share as F41-F43 for M711 (Section 3.4)	sbs_na_1a_se_r2
M72 – Scientific R&D	100% M7211,	sbs_na_1a_se_r2
	0–100% M7219	
M73 - Market research	0–100% M732	sbs_na_1a_se_r2
M74 - Other scientific	0–100% M741, M749	sbs_na_1a_se_r2
M75 - Veterinary	100%	sbs_na_1a_se_r2
N77 - Rental and leasing	G46-G47 bio-based share for N7729 (Section 3.2)	sbs_na_1a_se_r2
	0–100% N7739	
N78 - Employment	0%	–
N79 - Travel agency	0%-(I551-I552) share (Section 3.3)	sbs_na_1a_se_r2
N80 - Security	0%	–
N81 - Landscape	100% N813	sbs_na_1a_se_r2
N82 - Business support	0%	–
O84 - Public administration	Minimum share=0%, maximum share from gov_10a_exp (Section 3.5)	nama_10_a64
P85 - Education	Eurostat educ_uoe_fine04, educ_uoe_perp02, educ_uoe_grad02 (section 3.4)	nama_10_a64
Q86-Q88 – Health, social	0%	–
R90-R92 - Art, culture	Minimum share=0%, maximum share from cult_emp_n2 (Section 3.3)	nama_10_a64
R93, S94 - Sport, organis.	0–100%	nama_10_a64
S95 - Repair	Sector bio-based share of the product repaired*0–100% S9525 (Section 3.2)	sbs_na_1a_se_r2
S96 - Personal service	0%	nama_10_a64
T97-T98 – Household services	0–100%	nama_10_a64

Note: The NACE groups (3-digit NACE codes) and classes (4-digit NACE codes) that are not reported on the table do not belong to the bioeconomy (i.e., b_m,c,_*_y_* = 0%). The glossary at supplementary material S1 provides the full label of the activities represented here by their NACE code.

⁎ See Ronzon et al. (2020a).

When bioeconomy services cannot be clearly delimited in available statistics, a low and a high estimation of b_n,c,y_ or b_m,c,y_ is calculated, referred to as a minimum-maximum range in the text (see summary of assumptions in column 2 of Table 1 and details in Sections 3.2 to 3.6). Variables estimations are made as 3-year averages to reduce year-specific bias (i.e., 2008–2010 and 2015–2017), except for the computation of annual growth rates.

The following sections describe the assumptions underlying the quantification of b_n,c,y_ according to the different types of bioeconomy services identified at Section 2 (see also Fig. 1): "services associated with tangible bio-based goods" are addressed in Section 3.2, "natural environment-related services" in Section 3.3, “knowledge-based services in the field of the bioeconomy” in Section 3.4 and “support services for the development of bio-based markets” in Section 3.5.

### 3.2 Bioeconomy services associated with tangible bio-based goods

This category includes trade (G46-G47), transport (H49-H53), rental and leasing (N77) and repairing (S95) of bio-based products, food services (I56), publishing activities (J58) and some household services (T97_98). In the authors' view, these activities match the EU definition of using biological resources for the downstream production of a service. Following an output-based approach, we consider these services part of the bioeconomy to the extent to which the product they are associated with is of biological origin (i.e., bio-based output share b_n,c,_*_y_* = biomass content of the associated product). For instance, the b_m,c,y_ of wholesaling textile is equal to the biomass content of textile. Concretely:

• For trade, transport, rental and leasing and repairing: minimum and maximum b_n,c,y_ of bio-based products are taken from Ronzon et al. (2020b) and otherwise assumed unknown (0 <b_n,c,_*_y_* <1).
• For food services: b_n=I56,c,_*_y_* = 1 as the food output is fully (edible) biomass.
• For publishing activities b_m=J852,c,_*_y_* = 0 for software publishing since the output is virtual; 0 < b_m,c,_*_y_* < 1 for other publishing activities since publishing in print cannot be distinguished from electronic, audio or online publishing in available statistics (see NACE J58 4-digit codes in column 2 of Table 1).
• For household services: 0 < b_n=T97_98,c,_*_y_* < 1 as available statistics do not distinguish between the production of bio-based and other types of products from household activities.

### 3.3 Natural environment-related services of the bioeconomy

This category includes rural accommodation (I55), travel agency activities (N79), landscape service activities (N813) and cultural, (outdoor) sports and recreation activities (R90-R93). More specifically, it refers to the marketed services provided by land and marine ecosystems in the EU bioeconomy definition and those that are not accounted for in Section 3.2. Landscape service activities (N813) create or maintain ecosystems (e.g. planting, care and maintenance of parks and gardens) while the other NACE activities of this category are classified as beneficiaries of outdoor recreation services (La Notte et al. 2017, p. 70). It is worth noting that the common international classification of ecosystem services (CICES V5.1) distinguishes between biotic and abiotic outdoor cultural ecosystem services (including recreation services) (Haines-Young and Potschin 2018). However, the granularity of available statistics does not permit the singling out of the biotic part and therefore the quantifications consider by default the biotic and abiotic natural environment together.

Due to the intangible nature of the services of the present category, their bio-based output share cannot refer to their biomass content as for the previous category. They rather correspond to the use of or the valuation of (semi-) natural environments. Concretely:

• For accommodation services: b_n=I55,c,y_ is the proportion of nights spent in rural areas (100% natural environment) and towns and suburbs (arbitrarily set as 25–75% natural environments by the authors), using Eurostat (2020i) data at the NACE 3-digit level.
• For travel agencies: b_n=N79,c,y_ is the combined bio-based output share of hotels and short-stay accommodations (I551-I552) in the absence of ad'hoc data.
• For services to building and landscape activities: b_n=N81,c,y_ is based on landscape service activities only (b_m=N813,c,_*_y_* = 1).
• For sport and recreation: 0 < b_n=R93,c,_*_y_* < 1, the proportion of outdoor sports activities being unreported.
• For arts, cultural and entertainment activities: within this aggregate only NACE R91 matches a bioeconomy definition (to an unknown proportion) with the sub-activities of libraries (the output 'printed books' are essentially bio-based), botanical and zoological gardens and nature reserves activities (natural environment related services). The maximum b_n=R90_92,c,y_ is the proportion of people employed in NACE R91 (Eurostat, 2020d data) on total R90_92 workers (Eurostat, 2020h data). The minimum b_n=R90_92,c,_*_y_* = 0 in the absence of better data.

The valuation of this class of bioeconomy services takes a focused methodology based on extracted ‘rents’ from said services by enterprises. Whilst this approach is consistent with the value added data from Eurostat's structural business statistics and national accounts, it ignores ethical considerations relating to accountable shared burdens that arise from the extraction of rents from natural ‘public goods’ (Capasso 2021, Section 3.1) including the pricing of access or even ‘polluter-pays’ type market externality corrections. This issue is further discussed in Section 4.4.

### 3.4 Knowledge-based services in the field of the bioeconomy

This category includes architecture and technical consultancy (M71), scientific activities (M72, M74), veterinary activities (M75) and education (P85). In the authors' view, it does not directly link with the EU definition of bioeconomy sectors as it neither refers to an ecosystem service nor to an activity that uses biological resources to produce a service. However, bioeconomy knowledge-based activities (e.g., in life sciences) are integrated into other bioeconomy initiatives. Notably, the bioeconomy concept first reached the EU policy agenda under the name of knowledge bio-based economy (KBBE) and the EU bioeconomy has mainly been supported by research and innovation policies (Birner, 2018; Patermann and Aguilar, 2018; Kardung and Wesseler, 2019). The output of knowledge activities cannot be measured with a biomass content, but rather by determining the proportion of knowledge or knowhow created or disseminated in bioeconomy-related disciplines. Concretely:

• For veterinary activities: b_n=M75,c,_*_y_* = 1 since they deliver knowhow in bioeconomy disciplines (e.g. zoology or husbandry) besides being based on life science knowledge. It could be argued that veterinary activities should be given the same treatment as any upstream activity of the manufacturing of bio-based products (e.g. the manufacturing of machinery) and thus not considered part of the bioeconomy from an output-based perspective. The authors however assume that veterinary activities not only provide an input to animal production but they also generate knowledge and knowhow in the life science domain, similarly to other scientific activities considered as “knowledge-based services in the field of the bioeconomy”.
• For architectural activities: b_n=M711,c,y=_b_n=_*_F_*_,c,y_, architectural activities are considered bio-based to the same extent construction activities are in Ronzon et al. (2021) which also follow an output-based approach.
• For scientific activities: scientific disciplines are classified as 100% bio-based, 0–100% bio-based and 0% bio-based (see NACE M72 and M74 3 and 4-digit codes in column 2 of Table 1).
• For education: the employment b_n=P85,c,y_ is the multiplication of the number of teachers and academics teaching at graduating level (Eurostat 2020c) with the proportion of graduates in bioeconomy fields (Eurostat 2020f). The value added b_n=P85,c,y_ is the multiplication of the proportion of public expenditure in graduating educational levels with the proportion of graduates in bioeconomy fields (Eurostat 2020f, 2020j). Graduates in bioeconomy fields are reported in Eurostat (2020f), considering that fully bioeconomy disciplines are biological sciences, food processing and agriculture, forestry, fisheries and veterinary. 0–100% bioeconomy-related disciplines are economics, political sciences, environment, earth sciences, statistics, environmental protection technology, electricity and energy, glass, paper, plastic and wood materials, textiles, architecture and building and civil engineering.

### 3.5 Support services for the development of bio-based markets

This category includes advertising and market research (M73), public administration (O84) and activities of membership organisations (S94). Activities in support of bio-based markets are sometimes accounted for as bioeconomy activities, although not explicitly in the EU bioeconomy strategy.

Available statistics do not indicate the relationship between market research and membership organisation with bio-based products or bioeconomy-related organisations. We therefore consider 0 < b_n,c,_*_y_* < 1 for these activities.

The bio-based output share of public administration corresponds to the proportion of activities realised in bioeconomy domains, such as the administration of programmes in support of the development of the bioeconomy or of bioeconomy sub-sectors (e.g., agricultural policy). Correspondence tables between the NACE classification and the classification of the functions of government (COFOG) (Eurostat 2011) only reveal a list of a few non bio-based 'functions of government', for which government expenditure are reported (see supplementary section S2). This leads to an over-estimate maximum b_n,c,y_ of employment in public administration with the ratio of compensation of employees that do not work in the non bio-based COFOGS over the total (Eurostat (2020 g, 2020e) data). A similar approach is undertaken for the calculation of the value added b_n=O84,c,y_ (see supplementary section S3 for more details). Both the employment and value added minimum are set to zero (b_n=O84,c,_*_y_* = 0).

### 3.6 Non bioeconomy services

The NACE divisions of services that do not fall into any of the categories described in Sections 3.2 to 3.5 are excluded from the bioeconomy scope (b_n,c,_*_y_* = 0% on Table 1).

## 4 Results

### 4.1 Economic and labour productivity growth in European bioeconomy services (2008–2017)

**Fig. 2:**
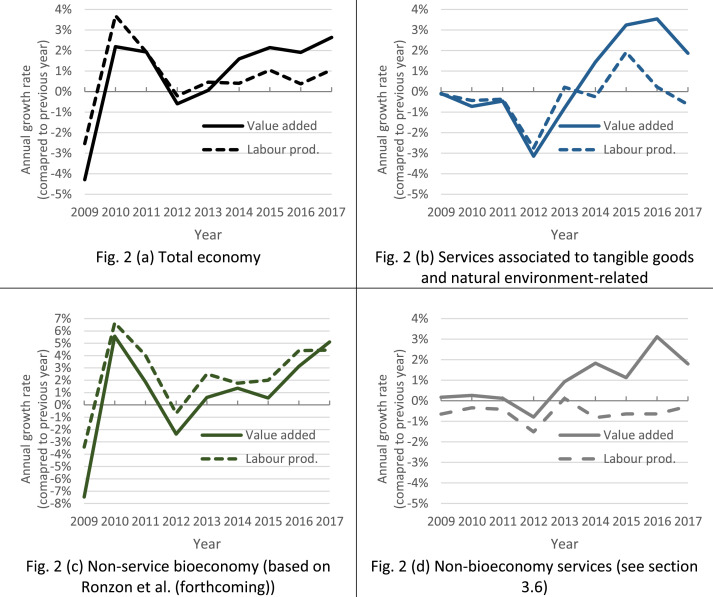
Annual growth rate of real value added and labour productivity for (a) the total economy and selected sub-sets of sectors, (b) the bioeconomy services directly linked to the EU definition, (c) the non-service bioeconomy sectors and (d) the services sectors that are not related with the bioeconomy. Note: Price index deflator based on the 2010 reference year (Eurostat nama_10_gdp). Calculations based on maximum bio-based output shares b_c,y,n_ for a major sector coverage.

To put our data into context, Fig. 2(a) shows the development of output (measured as real value added) and labour productivity (measured as real value added per person employed) growth for the total EU27 economy. The indicators swings coincide with two crises over the period of observation: the value added growth plunged to a −4.3% rate between 2008 and 2009 as a consequence of the 2008–2009 global financial crisis and again to −0.59% per annum after the 2010–2011 Euro area recession. Economic recovery happens after 2012 and value added stabilises between a 2%−3% annual growth rate at the end of the period (2015–2017). Labour productivity which is a factor of economic growth followed similar developments. Its contribution to value added growth is enhanced after 2012 when employment trends in the total number of persons employed becomes positive (not shown).

If we focus our attention on the categories of bioeconomy services directly linked to the EU (2018) definition, Fig. 2(b) shows that this aggregate of sectors has reacted differently to crises in the EU27. It has been less affected by the 2008–2009 financial crisis than the total EU27 economy aggregate but it has suffered from the general economic downturn. The crisis and its aftermath are characterised by a no-growth period in bioeconomy services (output and labour productivity growth varying between 0% and −1% per annum). Already vulnerable, bioeconomy services are then hit harder by the 2010–2011 Euro area recession and the indicators of output and labour productivity growth plunge by −3.1% and −2.7%, respectively, in 2012. Within this aggregate, the services associated to bio-based tangible goods deteriorate most (i.e. trade, transport, rental, repairing of bio-based products, food services, publishing activities and some household services).

After 2012, the total number of persons employed in bioeconomy services starts growing again and amplifies the positive effect of improving labour productivity on value added growth. Bioeconomy services flourish relatively to the whole economy: their value added growth is 0.7 percentage points higher than in the total economy (2015–2017). Interestingly, this observation can be generalised to 21 of the 27 EU Member States (not shown). Maximum differences are observed in Portugal and Romania where value added growth is 4.6 and 6.1 percentage points higher in bioeconomy services than in total national economy. At the other extreme, a difference of −8.8 and −3.0 percentage points is observed in Ireland and Estonia.

Similar trends also hold for a broader coverage of bioeconomy services (i.e., by including the bioeconomy knowledge-based services and the services in support to bio-based markets). They are consistent with Van Ark and Jäger (2017)’s observation that the EU services sector (bioeconomy and non-bioeconomy included) tended to increase its importance in the economy, and to recover slightly better from the crisis.

Compared to the non-bioeconomy services performance in Fig. 2(d), the aggregate of bioeconomy services in Fig. 2(b) reacted more negatively to the Euro area recession (−3.14% vs. −0.79% of value added growth between 2011 and 2012) but they demonstrated capacity for improving labour productivity in the following years. In contrast, in non-bioeconomy services, the negative labour productivity growth since 2013 has acted as a break on their value added growth.

Finally, from a quick comparison with the non-service bioeconomy's dynamic shown in Fig. 2(c), one cannot clearly discern whether bioeconomy services follow or lead the rest of the bioeconomy. They have been more resilient to the 2008–2009 crisis but they underwent a similar negative value added growth rate in 2012. Both aggregates of sectors recover at a similar pace immediately after 2012. However, after 2015 bioeconomy services’ growth has been limited by its reduced labour productivity while non-service bioeconomy value added growth is driven by its increasing labour productivity.

### 4.2 Bioeconomy services within the EU economy

The dynamic of bioeconomy services is largely influenced by their relative size within the broader economy and by their economic structure. Our calculations confirm the significant share of bioeconomy services within the EU27′s economic activity, whilst further suggesting that this influence is growing over time. Indeed, if we restrict the scope of bioeconomy services to the services associated to tangible goods or related to the natural environment (EU definition), we find that they contributed between 5.0–8.6% of EU27 GDP and between 10.2–16.9% of the EU27 labour force on average between 2015 and 2017 (i.e., €563-€967 billion annual value added and 20–33 million workers, Table 2). Comparing 2017 with 2008 highlights economic growth and employment creation. More specifically, the collective of bioeconomy services created between €68 and €89 billion of additional annual value added, and between 1.37 and 1.38 million additional workers. This has resulted in per worker labour productivity gains of between €1000 and €2000. However the aggregate labour productivity of bioeconomy services remains slightly below non-service bioeconomy labour productivity (approximatively €30,000 value added per worker vs. €35,000 per worker in Ronzon et al. (2021)).

**Table 2:** Value added, employment and labour productivity in bioeconomy services in 2008–2010 and 2015–2017 (3-year averages, output approach).

	Value added(billion €)				Number of people employed(thousand)				Labour productivity(thousand € per person employed)			
	av. 2008–2010		av. 2015–2017		av. 2008–2010		av. 2015–2017		av. 2008–2010		av. 2015–2017	
	min	max	min	max	min	max	min	max	min	max	min	max
**Associated with tangible bb goods**	**452**	**752**	**511**	**813**	**16,918**	**27,704**	**18,116**	**28,612**	**27**	**27**	**28**	**28**
G46 - Wholesale	138	226	155	244	2,787	4,697	2,826	4,584	50	48	55	53
G47 - Retail trade	113	182	125	198	5,370	8,469	5,452	8,488	21	21	23	23
H49 - Land transport	27	43	30	47	936	1,506	1,010	1,595	28	29	30	30
H50 - Water transport	4	7	4	6	36	60	34	53	114	114	110	113
H51 - Air transport	–	–	–	1	4	6	4	6	58	58	104	105
H52 - Warehousing	39	64	45	71	727	1,173	805	1,272	54	55	56	56
H53 - Postal activities	14	22	14	22	509	821	520	821	27	27	26	26
I56 - Food services	115	115	135	135	6,475	6,475	7,382	7,382	18	18	18	18
J58 - Publishing	–	33	–	29	–	622	–	549	–	53	–	52
N77 - Rental and Leasing	1	15	2	15	26	136	33	168	51	108	50	88
S95 - Repairing	1	1	1	1	50	86	50	85	15	15	15	15
T97_98 - Households services	–	44	–	44	–	3654	–	3608	–	12	–	12
**Natural environment-related**	**42**	**125**	**52**	**154**	**1,407**	**3,489**	**1593**	**3,946**	**30**	**36**	**32**	**39**
I55 - Accommodation	23	32	28	40	830	1,142	920	1272	27	28	31	31
N79 - Travel agency	7	10	8	12	182	246	185	253	41	41	46	46
N81 - Landscape activities	12	12	15	15	396	396	488	488	29	29	30	30
R90_92 - Libraries and cultural	–	22	–	24	–	493	–	509	–	44	–	48
R93 – Sport and recreation	–	50	–	63	–	1212	–	1423	–	41	–	44
**Bioeconomy knowledge-based**	**22**	**86**	**26**	**109**	**470**	**1,956**	**549**	**2,205**	**46**	**44**	**48**	**50**
M71 - Architecture	6	7	7	8	123	130	132	139	51	51	56	56
M72 - Scientific R&D	2	18	3	29	44	394	63	454	53	46	52	64
M74 - Other scientific service	–	23	–	30	–	611	–	738	–	38	–	41
M75 - Veterinary	5	5	7	7	155	155	196	196	34	34	34	34
P85 - Education	8	33	9	36	148	666	158	678	53	49	58	53
**Bioeconomy support services**	**–**	**472**	**–**	**531**	**–**	**8,579**	**–**	**8,668**	**–**	**55**	**–**	**61**
M73 - Market research	–	7	–	6	–	153	–	136	–	43	–	44
O84 - Public administration	–	403	–	448	–	6,476	–	6,463	–	62	–	69
S94 – Membership organisations	–	63	–	77	–	1,950	–	2,068	–	32	–	37
**Bioeconomy services**	**516**	**1,435**	**589**	**1,607**	**18,795**	**41,728**	**20,258**	**43,429**	**27**	**34**	**29**	**37**

Three sectors account for more than 60% of the value added and employment in EU bioeconomy services (EU definition) considering both minimum and maximum estimates, namely wholesale and retail trade of bio-based products, and the food and beverage service activities. Food services were an engine of employment growth and the main contributors to value added increases (net employment rise of 900,000 and +€20 billion annual value added in the decade up to 2017, Table 2). The evolution of retail trade of bio-based products has been more stable while wholesale activities employed 100,000 persons less at the end of the observed period according to our maximum estimate (vs. +18,000 according to our minimum estimate).

Accounting for less than 7% of total bioeconomy services value added and employment in 2017 (EU definition), sport and recreation activities strongly contributed to growth (+€14 billion value added, +211,000 workers in maximum estimates). Accommodation also played a strong employment role, ending with around 90,000–130,000 additional workers at the end of the period.

Note that bioeconomy knowledge-based services and bioeconomy support services would add 0.2–5.7% of GDP and 0.3–5.6% of total employment. The latter are not well represented in our calculations and not explicitly mentioned in the EU bioeconomy strategy.

### 4.3 Bioeconomy services in EU Member States' economies

The contribution of services that unambiguously qualify for the EU bioeconomy strategy definition (i.e., services associated to tangible goods or related to the natural environment) varies across EU Member States between 3.0% and 14.5% of GDP and between 5.8 and 27.6% of total employment.

From a sectoral perspective, similar to the EU27 picture commented in Section 4.2, the wholesale and retail trade of bio-based products are strong pillars of the bioeconomy in EU Member States, contributing each to more than 10% of the total bioeconomy service value added and employment in all Member States (supplementary section S4). Food and beverage service activities also play a strong employment role (15–47% total employment except in Portugal with 10–18%) and provide more than 10% of bioeconomy services' value added in almost all Member States.

**Fig. 3:**
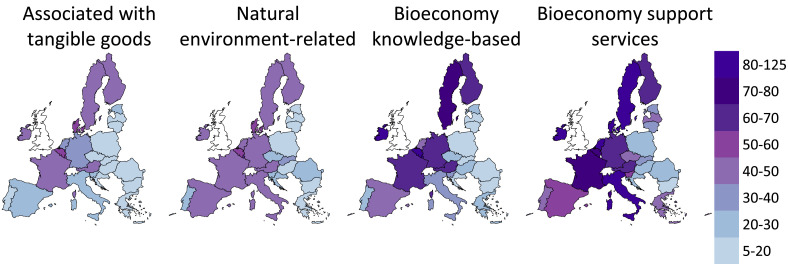
Labour productivity of EU Member States' bioeconomy services in €1000 per persons employed (maximum estimates, 2015–2017) Note: Minimum estimates are not shown as they convey messages similar to maximum estimates.

Fig. 3 maps the amount of value added generated per worker in the four categories of bioeconomy services quantified in this study (maximum estimate, 2015–2017). A gradient of labour productivity is visible from left to right with services associated with bio-based products being the least labour productive and bioeconomy supporting services being the most productive (columns 1 and 2 in Fig. 3).

Aside from the heterogeneous outcomes across EU Member States, the map highlights an East-West difference in labour productivity performance. On the one hand, the bioeconomy services of Bulgaria, Croatia, Hungary, Poland and Romania remain below €30,000 per person employed in all four types of services. The same happens in Baltic countries, Czechia, Greece and Portugal if one excludes bioeconomy support services. On the other hand, labour productivity exceeds €40,000 per worker in services related to bio-based products and with the natural environment in Austria, France, Finland and Sweden and exceeds €50,000 per worker in Belgium, Denmark and Luxembourg. In all these Member States plus Germany and Ireland, labour productivity of knowledge and bioeconomy support services is above €60,000 per worker.

### 4.4 Discussion of the results and the methodology

To the best of the authors’ knowledge, there is no other published attempt to quantify the size of bioeconomy services in Europe employing an output-based approach. A principle observation is that the estimates presented here – i.e., €589-€1607 million of value added and 19–42 million workers in the broad bioeconomy definition – are far higher than with the input-based approach presented in Kuosmanen et al. (2020, Section 4) which estimate approx. €370 billion of value added and 8.5 million workers in the EU28 bioeconomy services sectors of NACE G to T divisions. The difference arises from a low use of bio-based inputs into total inputs by the services industries. Indeed the bio-based input shares are inferior to 8% in NACE G to T sectors in the above-cited study (EU28, 2015). The only exception is accommodation and food services with a bio-based input share of 35% for the EU28 (2015), which nevertheless remains below the bio-based output share quantified in the present study (i.e., 39–55% for accommodation and 100% for food services in EU27 (2015)). The split between service and non-service bioeconomy sectors following the approach by Cingiz et al. (2021) is not publically available. In any case, direct comparison with our output-based quantification would also be hampered by methodological differences.

Ronzon et al. (2021) quantifies value added and employment in biomass producing and biomass manufacturing activities (the non-service bioeconomy) also following an output-based approach. Interestingly, our most conservative quantification (i.e., the minimum range) of bioeconomy services’ value added is comparable in size with the value added of the non-service bioeconomy quantified by Ronzon et al. (2021). The employment size of bioeconomy services, measured in number of workers, is even bigger than the one of the non-service bioeconomy (same source). In sum, the integration of bioeconomy services into the updated EU bioeconomy strategy more than doubles the value added and employment size of the bioeconomy compared to the initial strategy definition.

**Fig. 4:**
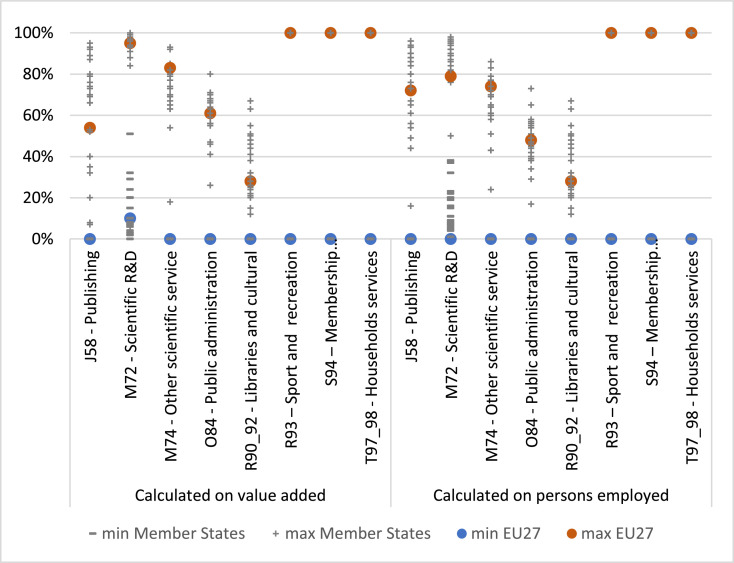
Ranges of employment (E) and value added (VA) shares (b_n,c_) for the bioeconomy services with a difference higher than 25% between their minimum and maximum b_n,c,y_ in the EU27 (*y*=average 2015–2017), and EU Member States distribution. Notes: b_n,c,_*_y_*_=av.2015–2017_ are represented with large blue (for minimum) and orange (for maximum) squares for the EU27, and with `-' (minimum b_n,c,_*_y_*_=av.2015–2017_) and `+' (maximum b_n,c,_*_y_*_=av.2015–2017_) points for the EU Member States. For example, calculated on value added (VA) data, 0%<b_n=J58,_*_y_*_=av.2015–2017_<54% for the EU27 (large blue and orange squares) and 0%<b_n=J58,_*_y_*_=av.2015–2017_<95% in Member States (distribution range of `-' and `+' points).

The choice of methodology and associated assumptions does, however, give rise to uncertainty in the estimates. The precision of the estimates is highly dependant on the level of granularity with which bioeconomy fields of activities are represented in official statistics. In the best case, some bioeconomy services or sub-activities can be directly based on Eurostat structural business statistics or national accounts databases and compiled as such in our study (b_n,c,_*_y_* = 100% in Table 1). In the worst case, the broadness of the NACE categories and the scarcity of detailed data from other official statistics permitting their disaggregation at the Member State level, impedes the determination of bio-based output shares (0 < b_n,c,_*_y_* < 1 or 0 < b_m,c,_*_y_* < 1 in Table 1). Supplementary section S5 summarises the dispersion of bio-based output shares for the period 2015–2017 (3-year averages) by bioeconomy service sector *n* using the value added (VA) or employment (E) data source for calculation. Fig. 4 highlights those NACE sectors that show a difference higher than 25% between the minimum and maximum EU share (coloured dots), both when calculated in value added and in employment terms. These sectors are publishing (J58), scientific activities (M72 and M74), public administration (O84), cultural, (outdoor) sports and recreation activities (R90-R93), membership organisations (S94) and household services (T97_98). In three of these sectors (R93, S94 and T97_98), only the maximum shares (b_n,c,y_) could be quantified, whilst the minimum shares were set to zero.

**Fig. 5:**
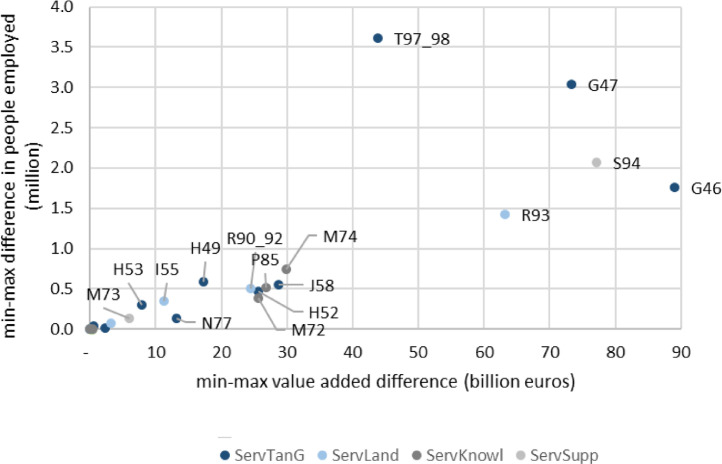
Uncertainty chart associated to the quantification of the value added (x-axis) and employment (y-axis) in bioeconomy services in the EU27 (difference between the minimum and maximum quantification, 3-year averages 2015–2017). Note: The position (447; 6.5) of O84 is out of scale.

Fig. 5 provides an uncertainty chart that reports the difference between the minimum and maximum value of value added (x-axis) and employment (y-axis) quantified for bioeconomy services. The sectors with the biggest minimum-maximum ranges both for employment and value added are positioned at the top-right corner. Among them, wholesale and retail trade activities (*n* = G46 and G47) were not identified above among the sectors with a weak representation of their bioeconomy sub-activities in European statistics. Their large economic and employment size implies that slight variations in bio-based shares can result in large variations in their bioeconomy size. Interestingly, three of the five sectors in the top-right corner are "bioeconomy services associated with tangible bio-based goods". Positioned in the bottom left corner, water and air transport (H50, H51), architectural and engineering activities (M71), veterinary activities (M75), travel agencies (N79) and repair of household goods (S95) are quantified with more precision.

Some methodological assumptions made in this paper are still a matter of debate and could be considered differently in other contexts or by other stakeholders. It was mentioned in Section 3.4 that veterinary activities (M75) is given the same treatment as the scientific activities classified under the NACE codes M72 and M74, considering that all three categories deliver knowledge and knowhow in the domain of life science. As a result, the employment and value added generated by veterinary activities is included as part of the bioeconomy's performance. Another approach could have been to consider veterinary activities as an input provider to agriculture (A01) to the same extent as any other non-biomass input provider (e.g. machinery). Under that alternative assumption, veterinary activities would not have contributed to the bioeconomy's performance. This particular point of debate demonstrates that the delimitation of the bioeconomy's scope remains under scrutiny.

The distinction between services provided by natural versus biological environments is another point of debate. While Finnish statistics on the bioeconomy include the employment and value added generated by non-bio renewable energies (e.g. from wind or solar resources in Luke (2020)), the present paper takes a stricter interpretation of the bioeconomy where only biological-based services are included. The only exception lies in the category of services termed “natural environment-related services of the bioeconomy” where available statistics do not enable us to exclude the beneficiaries from non-biological natural environments (e.g. frozen or rocky or geothermal areas with limited presence of living beings). Despite this potential bias, we expect the latter to play a non-significant role within the overall service bioeconomy.

Finally, there is the issue of regulating the management and protection of natural environments through the correction of market externalities that commonly arise from the use of public goods. For example, private beneficiaries of these natural areas such as hotels, campsites (I55) and providers of outdoor sports and recreation activities (R90-R93) may well play an active role in the preservation of the recreational ecosystem services, either directly by participating to local maintenance programs, or indirectly via payment for ecosystem services programs or political engagement. But they also may contribute to the degradation of the natural areas on which their business depends (Capasso 2021, Section 3.1). Examining the issue of market regulation, Mauerhofer et al. (2013), posit the use of polluter pays principle and payments for ecosystem services market instruments as complementary measures for internalising the capital costs of such ecosystem services. Moreover, in a bid to increase transparency, further initiatives seek to place monetary value on ecosystem services through the establishment of ecosystem accounts that are compatible with the classical national accounts (e.g. the United Nations’ System of Environmental Economic Accounting (SEEA) or the European Integrated system of Natural Capital and ecosystem services Accounting (INCA)). According to the latest edition of the European accounting for ecosystem services, the value of recreational ecosystem services in the EU28 is as high as €80.3 billion (Vysna et al., 2021). The integration of the value of ecosystem services to the total value generated by the bioeconomy represents an important research avenue and would better reflect the EU definition of the bioeconomy that explicitly includes the services provided by land and marine ecosystems.

## 5 Conclusion

Employing a mix of policy insight and Eurostat statistics, this study tests an output-based methodology for measuring and monitoring the value added and employment size of bioeconomy services. Despite uncertainty issues, our results highlight the growth potential and the considerable size of the bioeconomy services sector. Excluding the crises and post-crisis periods and judging on the most stable part of our period of observation (2015–2017), the aggregate of bioeconomy services exhibited stronger value added and labour productivity growth than the total economy aggregate at the EU27 level and in a large majority of Member States. The bioeconomy services directly matching the recently extended EU definition account for 5.0–8.6% of the EU GDP and 10.2–16.9% of the EU labour force. The lower range estimate is comparable in size with the non-service bioeconomy as quantified with a similar methodology (Ronzon et al., 2021). The maximum range is almost double the value added size and more than double the employment size of the non-service bioeconomy in that study. These proportions are likely to grow and labour productivity to improve in the future if past trends continue.

In the context of the Green Deal and the Recovery plan, the study highlights potential for growth and employment creation in specific bioeconomy service sectors. Three sectors play a pivotal role and account for more than 60% of the value added and employment in EU bioeconomy services: the wholesale, the retail trade of bio-based products and the food and beverage service activities. Food and beverage services are found to have been the main source of employment and value added creation amongst the services matching the authors’ interpretation of the European Commission definition of bioeconomy services. To a lesser extent, employment creation has also been strong in accommodation and sport and recreation activities. Looking at labour productivity as a source of economic growth, the study finds the two categories of bioeconomy services directly linking with the EU Bioeconomy strategy (i.e., the services associated to tangible bio-based goods and the natural environment-related services) are also the least labour productive bioeconomy services of the four categories identified in this study (the other two being the knowledge-based and market support services). Moreover, cross-country comparisons stress the existence of an East-West difference in labour productivity of bioeconomy services which confirms the usefulness of tailored geographical bioeconomy initiatives in support of levelling-up initiatives, as initiated with the Horizon 2020 CSA-BIOEASTup.

Another outcome of this study is to identify key data knowledge gaps, thereby providing orientation for an improved representation of bioeconomy service activities within national and European statistical frameworks. The knowledge-based services in the field of the bioeconomy and the services in support of the development of bio-based markets suffer from the largest uncertainties in the determination of their bio-based output share (in particular for scientific research, education, public administration and activities of [bioeconomy] membership organisations). The services directly targeted by the EU bioeconomy strategy are reported with more detailed information in European statistics (i.e., the services associated with tangible bio-based products and natural environment-related services) than the other two categories of upstream bioeconomy services. However, in some cases (e.g., rental and leasing, sport and recreation and households service activities), the bioeconomy component remains hidden.

Eurostat's business statistics provide (4-digit) disaggregated information on wholesale and retail trade activities but the large economic and employment size of these sectors implies that even slight variations in the quantification of their bio-based output share could result in large variations in the quantification of their bioeconomy size. The ongoing revision of the NACE classification and the foreseen revision of the statistical classification of products by activity (abbreviated CPA) consider stakeholder consultations and therefore constitute opportunities for the inclusion of more bioeconomy-specific codes. In the medium term, this process will help capture a more precise picture of European bioeconomy services and consequently strengthen bioeconomy monitoring frameworks and bioeconomy policy design processes.

Finally, whilst this study sheds light on the economic performance of bioeconomy services, interpreting a growing bioeconomy size as a policy objective per se would not be correct. The socio-economic dimension of bioeconomy services has to be assessed jointly with a comprehensive framework of indicators that reflect stakeholder perceptions of the term "sustainability" (Egenolf and Bringezu 2019; Schweinle et al., 2020). For example, the growth of nature tourism, as any activity related with the natural environment, may lead to degradation of the natural environment and the alteration or destruction of landscapes and biological resources (Capocchi et al., 2019). It seems that such concerns are shared by the European Commission that has devoted the third pillar (out of three) of its bioeconomy action plan to the understanding of the ecological boundaries of the bioeconomy. The European Commission is also building up a comprehensive bioeconomy monitoring system in which socio-economic indicators as the ones quantified in this study will be reported and analysed jointly with physical, ecological, social and innovation indicators.

## Authors’ statements

Neither this manuscript nor any parts of its content are currently under consideration or published in another journal. All the authors have approved the manuscript and agree with its submission. There are no conflicts of interest to declare.

The views expressed are those solely of the authors and should not in any circumstances be regarded as stating an official position of the European Commission.

## Appendix Supplementary materials

Image, application 1
