# Supplementary material for: An output-based measurement of EU bioeconomy services: Marrying statistics with policy insight
Source: Struct Chang Econ Dyn. 2022 Mar;60:290–301. doi: 10.1016/j.strueco.2021.10.005 (PMC8939855; doi:10.1016/j.strueco.2021.10.005)
Supplement: Supplementary file 1 [file mmc1.pdf]

SUPPLEMENTARY MATERIAL

S1 – Glossary of NACE sectors quoted in the manuscript

NACE revision 2 code	Label
A01	Agriculture and hunting
A02	Forestry
A03	Fishing
G45	Trade and repair of motor vehicles, etc.
G46	Wholesale trade (excl. motor vehicles, etc.)
G47	Retail trade (excl. motor vehicles, etc.)
H49	Land transport
H50	Water transport
H51	Air transport
H52	Warehousing and support activities for transportation
H53	Postal and courier activities
I55	Accommodation
I551	Hotels and similar accommodation
I552	Holiday and other short-stay accommodation
I553	Camping grounds, recreational vehicle parks and trailer parks
I56	Food and beverage service activities
J58	Publishing activities
J5811	Book publishing
J5812	Publishing of directories and mailing lists
J5813	Publishing of newspapers
J5814	Publishing of journals and periodicals
J5819	Other publishing activities
J59	Motion picture, video and television programme production, sound recording and music publishing activities
J60	Programming and broadcasting activities
J61	Telecommunications
J62	Computer programming, consultancy and related activities
J63	Information service activities
K64	Financial activities
K65	Insurance activities
K66	Activities auxiliary to financial and insurance activities
L68	Real estate activities
M69	Legal and accounting activities
M70	Activities of head offices; management consultancy activities
M71	Architectural and engineering activities, etc.
M711	Architectural and engineering activities and related technical consultancy
M72	Scientific research and development
M7211	Research and experimental development on biotechnology
M7219	Other research and experimental development on natural sciences and engineering

M722	Research and experimental development on social sciences and humanities
M73	Advertising and market research
M732	Market research and public opinion polling
M74	Other professional, scientific and technical activities
M741	Specialised design activities
M749	Other professional, scientific and technical activities n.e.c.
M75	Veterinary activities
N77	Rental and leasing activities
N7721	Renting and leasing of recreational and sports goods
N7729	Renting and leasing of other personal and household goods
N7739	Renting and leasing of other machinery, equipment and tangible goods n.e.c.
N774	Leasing of intellectual property and similar products, except copyrighted works
N78	Employment activities
N813	Landscape service activities
N79	Travel agencies, etc.
N80	Security and investigation activities
N81	Services to buildings and landscape activities
N82	Office administrative, office support and other business support activities
O84	Public administration and social security
P85	Education
Q86	Human health activities
Q87_88	Social work activities
R90_92	Cultural activities and gambling
R93	Sport, amusement and recreation activities
S94	Activities of membership organisations
S95	Repair of household goods
S9523	Repair of footwear and leather goods
S9524	Repair of furniture and home furnishings
S9525	Repair of watches, clocks and jewellery
S9529	Repair of other personal and household goods
S96	Other personal service activities
T97_98	Household service activities

S2 – List of 0% bio-based COFOGS used for the quantification of $b_{n,c,y}$ for n= O84

COFOG99	COFOG99_LABEL
GF0106	General public services n.e.c.
GF0107	Public debt transactions
GF0108	Transfers of a general character between different levels of government
GF0201	Military defence
GF0202	Civil defence
GF0205	Defence n.e.c.
GF0301	Police services
GF0302	Fire - protection services
GF0303	Law courts
GF0304	Prisons
GF0306	Public order and safety n.e.c.
GF0602	Community development
GF0606	Housing and community amenities n.e.c.
GF0701	Medical products, appliances and equipment
GF0706	Health n.e.c.
GF1009	Social protection n.e.c.

S3 –Quantification of the maximum $b_{n,c,y}$ for n= O84, calculated on value added data

In the European System of Accounts (ESA), "The output of government (…) is equal to the sum of its costs of production [of which the compensation of employees]" (Eurostat 2013, paragraph 20.107). Therefore, the value added of NACE O84 can be reduced by the compensation of employees in the same non bio-based related COFOGS as reported at the supplementary section S4 for an over-estimation of the maximum $b_{n=O84,c,y}$ of public administration's value added.

More concretely, in the ESA:

- **Output** (O)= Compensation of employees (CE) + Intermediate consumption (IC) + Consumption of fixed capital (CFC) + Taxes (T) <u>with T≈0 in governmental sectors</u>

Therefore O ≈ CE + IC + CFC

- **Value Added** (VA) = O – IC

If we substitute O by the former equation: VA ≈ CE + IC + CFC – IC

IC cancels and: VA ≈ CE + CFC

In conclusion, if we deduce the CE from non bio-based related COFOGS (Eurostat (2020e) data) in VA (Eurostat (2020g) data), we have an estimation of the bio-based value added.

S4 – Sectoral contribution of bioeconomy services to total GDP and national labour force (EU27 and MSs, 2015-2017 3-year average)

S4.1. Sectoral contribution of the services that qualify to the EU definition to total GDP (minimum-maximum %, 2015-2017). Cells with a minimum contribution ≥10% are highlighted in light grey, cells with a minimum contribution ≥20% are highlighted in dark grey, cells with a minimum contribution ≥30% are written in red.

geo	G46	G47	H49	H50	H51	H52	H53	I55	I56	J58	N77	N79	N81	R90_92	R93	S95	T97_98
AT	23-24	19-21	5-6	.-.	0-0	6-7	2-2	12-16	15-24	0-2	0-3	1-2	1-1	0-2	0-4	0-0	0-1
BE	31-32	21-22	5-5	1-1	0-0	8-8	3-3	1-2	13-23	0-3	0-2	1-1	2-4	0-3	0-3	0-0	0-1
BG	37-40	18-19	10-10	0-0	0-0	7-8	2-2	6-6	9-14	0-1	0-1	1-1	0-1	0-3	0-3	0-0	.-.
CY	15-17	17-18	1-1	0-0	.-.	12-12	1-1	17-18	20-31	0-1	0-0	2-2	0-0	0-2	0-4	0-0	0-7
CZ	18-26	20-21	7-9	0-0	.-.	4-5	2-2	7-8	17-25	0-4	.-.	3-3	1-2	0-8	0-7	.-.	0-2
DE	25-28	21-22	4-4	1-1	0-0	8-8	3-3	4-4	12-22	0-4	0-2	2-2	3-5	0-1	0-7	0-0	0-3
DK	35-38	11-11	4-5	5-6	0-0	7-7	2-2	2-4	11-22	0-4	0-2	1-2	1-3	0-7	0-5	0-0	0-3
EE	25-27	16-18	8-9	0-0	.-.	14-15	1-1	4-6	10-20	0-3	0-4	1-2	1-1	0-4	0-6	.-.	0-1
EL	27-32	15-18	3-3	2-3	0-0	11-11	2-2	18-23	5-9	0-2	0-0	2-2	0-0	0-4	0-2	0-0	0-5
ES	22-27	19-22	5-6	0-0	0-0	8-10	1-1	5-5	15-25	0-2	0-1	1-1	1-1	0-2	0-9	0-0	0-8
FI	19-22	20-23	7-8	1-1	0-0	5-6	2-3	2-3	15-30	0-7	0-1	1-1	1-1	0-5	0-10	0-0	0-3
FR	22-22	23-25	5-5	0-0	0-0	7-7	3-3	3-4	18-29	0-3	1-2	1-1	2-3	0-2	0-8	0-0	0-2
HR	18-18	20-21	4-5	1-1	.-.	6-6	2-2	18-23	15-23	0-2	0-0	2-3	1-1	0-3	0-6	0-0	0-0
HU	28-32	22-24	9-10	0-0	0-0	11-12	3-3	4-4	8-13	0-3	0-1	1-1	1-1	0-4	0-5	0-0	0-0
IE	30-33	24-24	0-0	0-0	0-0	5-5	3-3	6-6	19-26	.-.	0-2	1-1	1-1	0-2	0-6	0-0	0-0
IT	21-24	20-24	5-5	1-1	0-0	8-9	2-2	5-6	15-26	0-2	0-1	1-1	1-1	0-2	0-5	0-0	0-13
LT	33-35	17-18	19-20	1-1	0-0	9-9	1-1	2-2	7-12	0-1	0-1	1-1	0-1	0-5	0-2	0-0	0-1
LU	37-40	16-17	5-5	.-.	.-.	.-.	.-.	3-4	21-32	0-0	0-3	.-.	2-3	0-2	0-5	0-0	0-5
LV	26-30	19-21	9-10	0-0	0-0	15-17	1-1	2-2	9-16	0-2	0-1	1-1	0-1	0-8	0-4	0-0	0-2
MT	20-25	21-23	2-2	.-.	.-.	.-.	.-.	15-15	17-32	0-1	0-0	3-3	.-.	0-15	0-4	0-0	0-3
NL	36-39	17-18	6-6	1-1	.-.	8-8	2-2	2-2	14-20	.-.	0-0	3-3	2-3	0-2	0-5	.-.	0-1
PL	33-38	26-27	10-11	0-0	0-0	6-7	2-2	2-3	6-10	0-3	0-1	1-1	1-1	0-3	0-4	0-0	0-2
PT	23-26	21-23	5-6	0-0	0-0	8-9	2-2	6-6	17-27	0-1	0-1	1-1	0-1	0-2	0-4	0-0	0-7
RO	35-39	23-25	9-10	0-0	0-0	6-6	2-2	3-3	8-13	0-1	0-1	1-1	1-1	0-7	0-4	0-0	.-.
SE	28-30	20-20	6-6	0-0	0-0	5-5	2-2	3-4	15-27	0-4	0-2	1-1	1-2	0-4	0-8	0-0	0-1
SI	21-22	20-20	10-10	0-0	0-0	9-9	2-2	9-11	14-23	0-3	0-0	2-2	0-1	0-4	0-3	0-0	0-1
SK	23-30	25-29	9-11	0-0	0-0	5-6	2-3	3-4	8-15	0-3	0-2	1-2	1-2	0-13	0-6	0-0	0-1
**EU27**	25-28	21-22	5-5	1-1	0-0	7-8	2-2	4-5	14-24	0-3	0-2	1-2	2-3	0-3	0-7	0-0	0-5

S4.2. Sectoral contribution of the services that qualify to the EU definition to total employment (minimum-maximum %, 2015-2017). Cells with a minimum contribution ≥10% are highlighted in light grey, cells with a minimum contribution ≥20% are highlighted in dark grey, cells with a minimum contribution ≥30% are written in red.

geo	G46	G47	H49	H50	H51	H52	H53	I55	I56	J58	N77	N79	N81	R90_92	R93	S95	T97_98
AT	13-14	22-27	4-5	0-0	0-0	2-3	1-2	12-16	27-38	0-1	0-1	1-2	1-2	0-1	0-3	0-0	0-1
BE	15-16	27-27	5-5	0-0	0-0	4-5	3-3	1-2	25-39	0-2	0-1	1-1	3-5	0-2	0-3	0-0	0-6
BG	18-19	33-34	8-8	0-0	0-0	4-4	2-2	5-5	20-28	0-1	0-0	1-1	0-1	0-2	0-3	0-0	.-.
CY	11-14	19-21	1-1	0-0	.-.	6-6	1-1	13-15	23-40	0-1	0-0	1-1	0-1	0-1	0-3	0-0	0-20
CZ	10-14	21-22	7-8	0-0	.-.	2-3	2-2	6-6	31-42	0-2	.-.	2-3	1-1	0-5	0-7	.-.	0-2
DE	11-11	26-26	3-3	0-0	0-0	5-5	4-4	4-5	24-40	0-2	0-1	1-1	3-4	0-1	0-4	0-0	0-12
DK	20-20	16-16	3-3	1-1	0-0	3-3	2-3	3-5	24-45	0-4	0-1	1-2	1-3	0-5	0-8	0-0	0-7
EE	15-16	25-25	9-9	0-0	.-.	6-7	2-2	5-6	19-33	0-3	0-1	1-2	1-2	0-5	0-7	.-.	0-0
EL	11-13	23-25	2-2	0-0	0-0	2-3	1-1	10-11	37-47	0-1	0-0	1-1	0-0	0-1	0-2	0-0	0-5
ES	15-17	23-27	4-5	0-0	0-0	3-4	1-1	3-3	26-40	0-1	0-0	1-1	1-2	0-1	0-5	0-0	0-15
FI	12-12	24-25	7-7	0-0	0-0	4-4	3-3	2-3	22-42	0-5	0-0	1-1	1-2	0-4	0-9	0-0	0-5
FR	14-14	28-29	5-5	0-0	.-.	3-4	4-4	3-3	22-36	0-2	1-1	1-1	2-4	0-2	0-9	0-0	0-5
HR	12-13	24-26	4-5	0-0	0-0	4-4	2-2	10-12	27-39	0-2	0-0	2-2	1-1	0-2	0-4	0-0	0-1
HU	16-17	31-32	8-8	0-0	0-0	6-6	3-3	3-3	19-28	0-2	0-1	1-1	1-2	0-4	0-4	0-1	0-1
IE	13-14	29-30	0-0	0-0	0-0	2-2	2-2	8-8	31-42	.-.	0-1	1-1	1-2	0-1	0-5	0-0	0-2
IT	11-13	21-27	3-4	0-0	0-0	4-5	2-2	3-4	21-41	0-1	0-0	1-1	1-2	0-1	0-3	0-0	0-30
LT	17-18	29-29	16-16	0-0	0-0	5-5	2-2	2-2	15-27	0-2	0-0	1-1	1-1	0-4	0-4	0-0	0-1
LU	15-17	20-22	6-7	.-.	.-.	.-.	.-.	3-5	32-50	0-0	0-0	.-.	2-3	0-1	0-4	0-0	0-11
LV	15-15	30-30	9-9	0-0	0-0	9-9	2-2	2-3	18-30	0-2	0-0	1-1	1-1	0-4	0-5	0-0	0-1
MT	16-16	27-28	2-2	0-0	.-.	6-6	1-1	9-11	26-37	0-1	0-0	2-2	.-.	0-2	0-2	0-0	0-2
NL	16-17	29-31	4-4	1-1	.-.	3-3	2-2	3-3	25-37	0-2	0-1	1-1	2-3	0-2	0-5	0-0	0-2
PL	21-21	36-37	11-11	0-0	0-0	4-4	3-3	2-3	10-18	0-2	0-0	1-1	1-2	0-3	0-4	0-0	0-1
PT	14-15	26-28	4-4	0-0	0-0	2-2	1-1	5-5	27-43	0-1	0-0	1-1	1-1	0-1	0-3	0-0	0-13
RO	22-24	32-33	11-11	0-0	0-0	4-4	3-3	3-3	16-23	0-1	0-0	1-1	1-2	0-1	0-2	0-0	.-.
SE	16-17	23-24	6-6	0-0	0-0	4-4	3-3	4-5	23-41	0-3	0-1	1-1	1-2	0-3	0-9	0-0	0-1
SI	13-15	20-21	8-9	0-0	0-0	3-3	2-2	9-11	25-38	0-2	0-0	2-2	1-1	0-4	0-4	0-0	0-2
SK	16-16	29-30	7-8	0-0	0-0	3-3	2-2	3-4	21-34	0-3	0-1	1-1	2-2	0-3	0-4	0-0	0-2
**EU27**	**14-14**	**26-28**	**5-5**	**0-0**	**0-0**	**4-4**	**3-3**	**4-5**	**23-37**	**0-2**	**0-1**	**1-1**	**1-2**	**0-2**	**0-4**	**0-0**	**0-11**

S5 – Ranges of employment (J) and value added (VA) shares ($b_{n,c}$) for the bioeconomy services in the EU27 (y=average 2015-2017), and EU Member States distribution

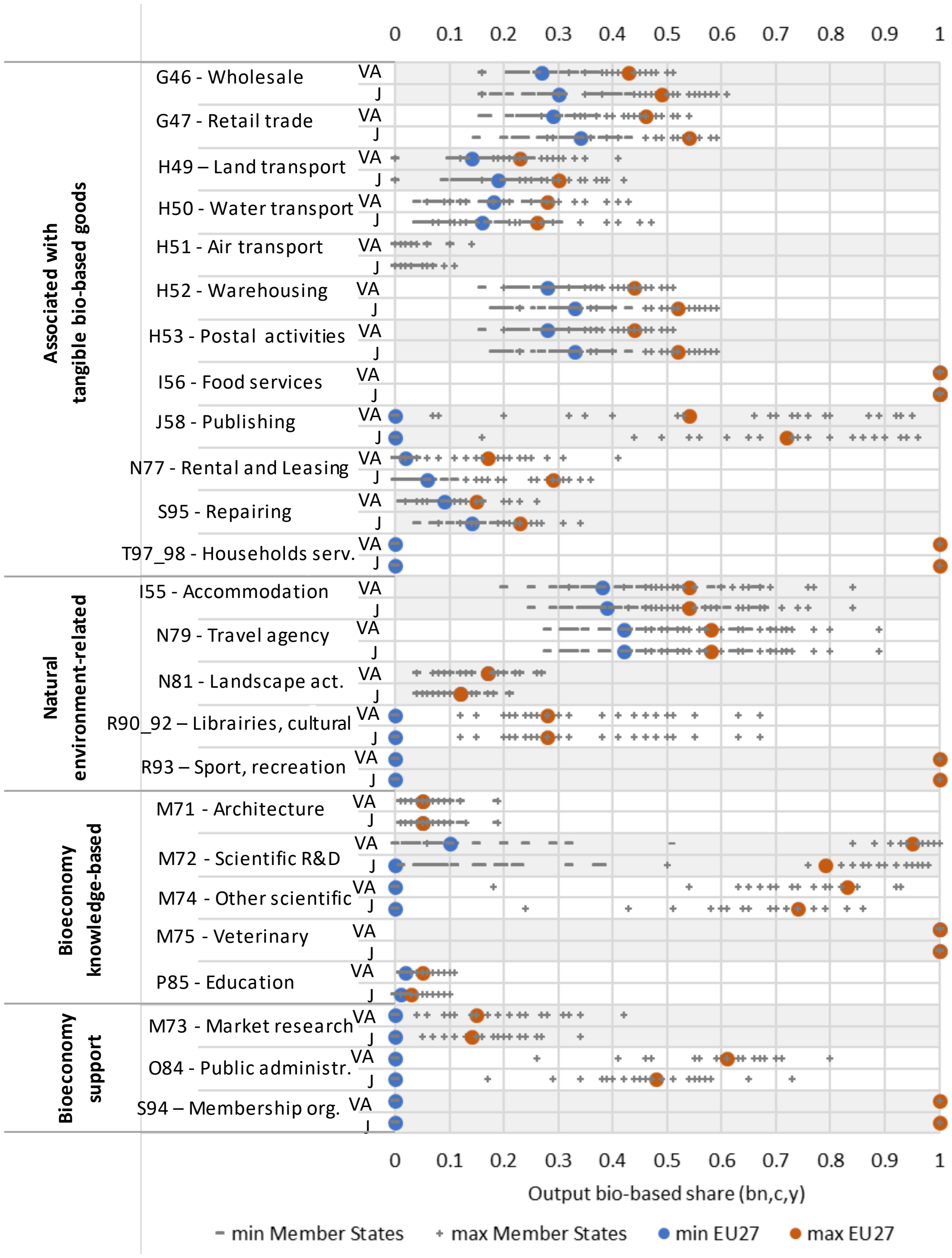

Notes: $b_{n,c,y=av.2015-2017}$ are represented with large blue (for minimum) and orange (for maximum) squares for the EU27, and with `-' (minimum $b_{n,c,y=av.2015-2017}$) and `+' (maximum $b_{n,c,y=av.2015-2017}$) points for the EU Member States. For example, calculated on value added (VA) data, 27%<$b_{n=G46,y=av.2015-2017}$<43% for the EU27 (large blue and orange squares) and 16%<$b_{n=G46,y=av.2015-2017}$<51% in Member States (distribution range of `-' and `+' points).
